# The Effect of Socio-Economic Status on Health Information Literacy among Urban Older Adults: Evidence from Western China

**DOI:** 10.3390/ijerph18073501

**Published:** 2021-03-28

**Authors:** Chengbo Li, Yanqi Guo

**Affiliations:** School of Journalism and communication, Chongqing University, Chongqing 400044, China

**Keywords:** health information literacy, health information literacy deficit, socio-economic status, social gradient, older Chinese adults

## Abstract

The present study aimed to observe the effect of socio-economic status on health information literacy and to identify whether there is a social gradient for health information literacy among urban older adults in Western China. This study employed a cross-sectional research design, and 812 urban participants aged 60 and older were enrolled in Western China. In the current study, only 16.7% of urban older adults reported having adequate health information literacy. Binary logistic regression analysis showed that socio-economic status factors including educational attainment, ethnicity, and financial strain were significantly and tightly associated with health information literacy. Additionally, other factors including suffering from chronic diseases, information-seeking activity, reading magazines and books, and watching television were also significantly linked to health information literacy. Consistent with existing studies, the findings indicate the health information literacy deficit and demonstrate the crucial impact of socio-economic status on health information literacy, which implies a social gradient in health information literacy. The importance of other factors related to health information literacy are discussed as well. The results suggest that reducing the health information literacy deficit and social gradient in health information literacy must be considered as an important priority when developing public health and health education strategies, programs, and actions among urban older adults in Western China.

## 1. Introduction

Health information literacy is defined as the ability to recognize a need for health information, to know how and where to find information about health, and to know how to evaluate and use the information to make good health decisions [1,2,3]. Previous studies of health information literacy mainly have been used in educational settings [4,5], health care settings [6], and in everyday life contexts [1].

Health information literacy has gained a growing importance on the health agenda around the world. First of all, health information literacy has a beneficial effect on health and well-being among people [7,8]. In addition, health information literacy is regarded as a panacea for poverty alleviation [9] among developing countries and is considered as an effective and appropriate tool of librarians [10] and educators [5] in particular. Moreover, health information literacy reinforcement as an important priority has been performed in developed countries such as the US [11] and the UK [12]. Especially, the “Better Information, Better Choices, Better Health” plan has been conducted in the UK [12].

As Eriksson et al. said “not everyone can master health information and be health-literate” [13]. There is evidence that older adults are considered to be the largest group with limited health information literacy [14,15,16] compared with younger adults. Thus, it is important to explore and understand the predictors of health information literacy and to establish ways and mechanisms of successfully promoting health information literacy among older adults.

## 2. Literature Review

### 2.1. Social Determinants of Health and Social Gradient in Health

In response to global health inequality, the World Health Organization set up the Commission on Social Determinants of Health in 2005 [17]. The social determinants of health (SDOH) consisted of the conditions of daily life, and those conditions referred to the distribution of income, power, goods, and services at global, national, and local levels and the circumstances in which people are born, grow, live, work, and age [18]. The social determinants of health are mostly responsible for the social gradient in health and the wide health disparities and inequities [18,19,20].

Social gradient in health means that people of lower socio-economic status have poorer health status and worse health outcomes, findings that have been well-documented in previous studies [21,22,23,24]. The social gradients in health run from the top to the bottom of the socio-economic spectrum [25], and they are often treated as health inequalities in socio-economic status [26] such as income, educational attainment, occupation [27,28,29], and ethnicity [30].

So far, previous studies have borne out claims that there is a social gradient in health behaviors, health risk behaviors, and health problems; for instance, someone who has lower social status is less likely to obtain healthcare services, such as dental attendance [31,32], and is more likely to adopt health risk behaviors, such as smoking [33], and consequently is more likely to suffer from a wide range of diseases including periodontal disease, depression, and obesity [34,35,36]. Moreover, the social gradient in health literacy has been confirmed in Europe [37]. However, few studies have confirmed the social gradient in health information literacy.

### 2.2. Factors Associated with Health Information Literacy

Many studies have focused on factors associated with health information literacy among older adults, with findings indicating five main factors: socio-economic status, demographic characteristics, health status, motivation, and sources of health information.

#### 2.2.1. Socio-Economic Status

Studies have illustrated that socio-economic status is a strong predictor of health information literacy. For instance, educational attainment is positively related to being health information literate, and better educated seniors always show more confidence in their ability to seek, understand, and master health-related information than those less educated [13]. Additionally, older adults who were doctors before retiring have higher health information literacy than those who were farmers [38]. Financial strain is an important determinant of health information literacy [38]. In addition, being a member of a racial or ethnic minority is related to a lower level of health information literacy among older adults [16].

#### 2.2.2. Other Factors

Significant evidence has demonstrated that some demographic attributes of older adults are associated with health information literacy. Age negatively impacts health information literacy among older adults. Older adults have fewer channels in which to obtain health information [13,39] and are less likely to be open to accepting online health information than younger adults [40]. Gender is a significant predictor of health information literacy among older adults [1,13]; however, some research found that the direction of the relationship between being female and health information literacy is unclear; only older men seem to perceive more usefulness of the Internet [41], and they often search for health information via the Internet [42].

Research found that older adults who report their health as being good or excellent are less likely to seek and use health information compared to their counterparts suffering multiple chronic illnesses [43,44,45,46,47]. Research has also demonstrated that motivation such as an interest in health information and information-seeking activity contribute to health information literacy [1]. It is not surprising that older adults who are more interested in health information and who are active at accessing it featured higher health information literacy [13]. Researchers found that sources of health information are strongly associated with health information literacy, particularly in the context of the Internet; more computer experience and more experience in seeking online health information contribute to more knowledge, expectations [48], and behaviors regarding decision-making in medicine and healthcare [49].

However, in spite of growing attention being paid to the predictors of health information literacy, information about the prevalence and predictors of health information literacy among urban older adults in Western China remains scare. Furthermore, it was discovered in the literature review that very few studies have been conducted to observe the effect of socio-economic status on health information literacy or to identify whether there is a social gradient for health information literacy.

### 2.3. Theoretical Framework

**Figure 1:**
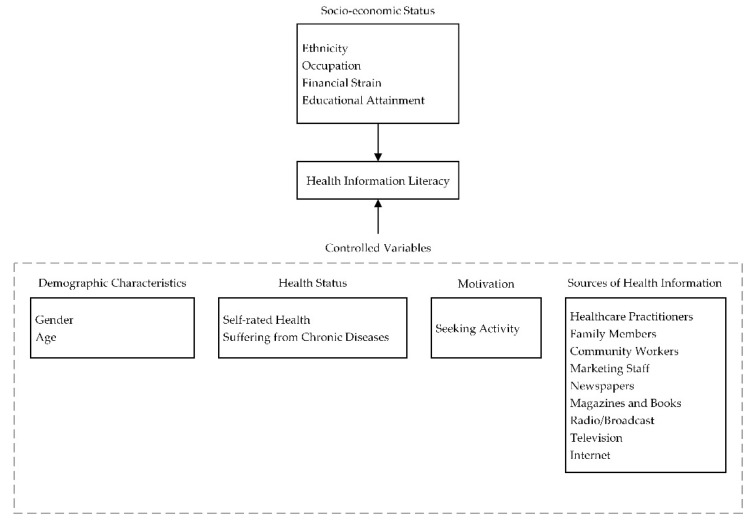
The conceptual model.

Based on the reviewed literature above, the theoretical framework in the present study is proposed as Figure 1.

## 3. Research Method

### 3.1. Data

This cross-sectional survey was conducted from July to September in 2017 in urban areas of Western China. Using a stratified multistage and cluster random sampling design, we randomly selected 812 urban older adults aged 60 and above who lived in Yinchuan City of the Ningxia Hui autonomous region, Wenshan city of Yunnan province, and the Yongchuan district of the Chongqing municipality in Western China.

First, three provinces or municipalities were selected in Western China. Ningxia was selected from North Western China, Yunnan and Chongqing were selected from South Western China. Second, within each province or municipality, one urban area was randomly selected. Yinchuan city was selected from Ningxia, Wenshan city was selected from Yunnan, and Yongchuan district was selected from Chongqing. Third, three blocks in urban areas were randomly selected within each city or district. Fourth, five urban residential communities were randomly selected within each block in Yinchuan city, 16 urban residential communities were randomly selected within each block in Wenshan city, 12 urban residential communities were randomly selected within each block in Yongchuan district. Fifth, households were randomly selected within each urban residential community in each province or municipality. In the case of households with more than one person aged 60 and older, one individual was selected at random using the Kish table. Therefore, 812 urban households were selected, the randomly selected households of Ningxia, Yunnan, and Chongqing were 203, 278, and 331, respectively. The participants with an in-person structured interview were provided written consent regarding the purposes and objectives of this study. The interviewees agreed to participate. A total of 890 questionnaires were issued and the response rate was 91.25% (812 of 890).

### 3.2. Measurement Instruments

Based on the literature review above, we examined potential predictors including demographic characteristics (gender, age), socio-economic status (educational attainment, occupation, financial strain, ethnicity), health status (self-rated health, suffering from chronic diseases), motivation (seeking activity), and sources of health information (healthcare practitioners, family members, community workers, marketing staff, newspapers, magazines and books, radio/broadcast, television, and Internet). These factors were selected because they were found to be associated with health information literacy among older adults in a substantial number of studies. We used a representative sample to understand the statistically significant relationship between these attributes and health information literacy among urban older adults in Western China.

#### 3.2.1. Health Information Literacy

Health information literacy was measured by the Chinese version of health information literacy questionnaire, which had been widely adopted and verified in previous studies [38,50,51,52] and could ensure reliability and validity. The health information literacy questionnaire consisted of 10 items [51] and was extracted from the Chinese version of the Citizen Health Literacy Questionnaire with 80 items [53]. More details about the 10 items of health information literacy and the 80 items of health literacy were presented in Nie et al. [51] and Li et al. [53], respectively.

The format of the 10 test items was in the form of questions of three types: single-answer questions tests (one score for a correct answer, zero score for an incorrect answer), multiple-answer questions tests (two scores for all correct answers, zero score for an incorrect answer), and situation questions tests in the form of reading comprehension about common information, instructions, and knowledge related to medicine and health in everyday life, which included single-answer questions and multiple-answer questions (with the same scoring criteria as the single or multiple-answer questions above). Especially, an “I don’t know” answer category was added to each item, and it was regarded as an incorrect answer with a score of zero. Following common practice for health information literacy measures [54,55], the range of the total score was 0–13, which was computed by adding scores of each item with equal weighting; the thresholds and ranges of different levels were defined according to assessment criteria of the required scores (80% of the total score). Hence, the resulting two levels were “inadequate” (0–9) and “adequate” (10–13) health information literacy.

#### 3.2.2. Socio-Economic Status

Socio-economic status included ethnicity (1 = Han, 2 = minority), educational attainment (1 = illiterate, 2 = primary school, 3 = junior high school, 4 = polytechnic school or senior high school, 5 = college and above), occupation (1 = ordinary staff, 2 = professional, 3 = manager, 4 = service industry employee, 5 = production staff, 6 = other), and financial strain, which was measured by a single item: “How do you assess your economic condition now?” Respondents rated their financial status on a 5-point Likert scale (1 = more than enough, 2 = good enough, 3 = approximately enough, 4 = somewhat difficult, 5 = very difficult).

#### 3.2.3. Other Variables

Demographic characteristics consisted of gender (0 = male, 1 = female) and age, which was divided into five categories: 1 = 60–64, 2 = 65–69, 3 = 70–74, 4 = 75–79, and 5 = 80+.

Health Status was measured in terms of self-rated health and suffering from chronic diseases. Self-rated health was measured by a single item: “How do you assess your health situation now?” Then respondents rated their health on a 5-point Likert scale (1 = very bad, 2 = bad, 3 = fair, 4 = good, 5 = very good). Suffering from chronic diseases was measured by another question: “Do you suffer from any chronic disease?” And the responses of respondents were recorded as yes or no; thus, each response was measured dichotomously (0 = no, 1 = yes).

As mentioned in Eriksson-Backa et al. [13], motivation was defined as seeking activity, which was measured by asking whether participants were more active at seeking health information, and the responses of respondents were dichotomized into yes or no; thus, each response was measured dichotomously (0 = no, 1 = yes).

Sources of health information were assessed using nine items (healthcare practitioners, family members, community workers, marketing staff, newspapers, magazines and books, radio/broadcast, television, and Internet). Participants were asked whether they had experienced certain sources for accessing and obtaining health information. Respondents were asked to rate each item on a two-point scale (0 = no, 1 = yes).

### 3.3. Analysis

The data analytic strategies for this study included descriptive statistics and logistic regression analysis. First, descriptive statistics were conducted to calculate the frequencies and percentages for all variables. Second, a binary logistic regression model was used to estimate the effect of the possible predictors on health information literacy, and the regression coefficients (B), Exp(B), confidence intervals (95% CI), and *p*-values were reported in this sample. IBM SPSS 22.0 software (SPSS Inc., Chicago, IL, USA) was used for all analysis. Statistical significance was set at 0.05.

## 4. Results

### 4.1. Characteristics of the Sample

As shown in Table 1, 812 participants aged 60 and older were selected from the urban areas of Western China, including 379 male respondents and 433 female respondents with a proportion of 46.7% and 53.3%, respectively. Moreover, only 16.7% of the total participants had an adequate level of health information literacy, whereas a vast majority of participants (83.3 percent) had an inadequate level of health information literacy.

Almost 90 percent of participants were aged at 60–79, and an overwhelming majority of participants (84.2 percent) were Han Chinese. Approximately half of participants (50.7 percent) had an education level that was either illiterate or primary school; specifically, 30.4% had primary education, whereas 20.3% were illiterate. Nearly 60% of respondents engaged in “production staff” work and reported financial strain status of “difficult” (17.6%) and “approximately enough” (41.1%). Over half of respondents rated their health status as being “good” (40.4%) and “very good” (11.6%). Most of the individuals (83.6%) were suffering from chronic diseases. In addition, around 65.9% of the participants were active at seeking health information; the proportions of the leading source including healthcare practitioners, television, family members were 86.3%, 55.9%, 54.7%, respectively, for accessing and obtaining health information.

### 4.2. Logistic Regression Model

A logistic regression model I—with all four socio-economic indicators introduced as independent variables—explained 11% of the variance of health information literacy (Table 2). Among the participants, educational attainment (B = 0.638, *p* < 0.001), financial strain (B = −0.454, *p* < 0.001), and ethnicity (B = −1.021, *p* < 0.01) were statistically and significantly associated with health information literacy. Specifically, participants of Han Chinese with a higher level of educational attainment and lower financial strain reported a higher level of health information literacy.

However, applying a new binary logistic regression model and controlling other factors, such as demographic attributes, health status, motivation variables, and sources of health information, we could conduct a better examination of the direct impact of the four socio-economic indicators on health information literacy as mentioned above.

Therefore, as shown in Table 2, the logistic regression model II was adjusted to include other factors to examine whether socio-economic status factors were still significantly associated with health information literacy. The variables used in the regression analysis explained 17.7% of the variance for participants in terms of health information literacy.

Among the participants, educational attainment (B = 0.531, *p* < 0.001), financial strain (B = −0.307, *p* < 0.05), and ethnicity (B = −1.003, *p* < 0.01) were still statistically related to health information literacy. Besides, suffering from chronic diseases (B = 1.082, *p* < 0.01), seeking activity (B = 1.025, *p* < 0.01), magazines and books (B = 0.949, *p* < 0.01), and television (B = 0.529, *p* < 0.05) were also significantly linked to health information literacy. Specifically, participants of Han Chinese with lower financial strain reported a higher level of health information literacy, whereas participants who had experienced certain sources including magazines and books and television for accessing and obtaining health information, those with higher levels of education, those with more chronic diseases, and those who were more active at seeking health information reported a higher level of health information literacy.

## 5. Discussion

Only 16.7% of the total participants had an adequate level of health information literacy in the current study, while the China Health Education Centre found that the national average of health information literacy among urban adults and older adults was 23.67% (aged 15–69) and 19.26% (aged 65–69) in 2012, respectively [56]. Meanwhile, a vast majority of participants (83.3 percent) had an inadequate level of health information literacy. As a result, the current research indicates a lower level of adequate health information literacy and a higher level of inadequate health information literacy among urban older adults in Western China in 2017, which implies that the health information literacy deficit is a challenge for public health in urban areas of Western China. This finding is consistent with a previous study that confirmed a health literacy deficit among the people in Europe [37]. Lower prevalence of health information literacy was also observed in previous studies conducted in other provinces in China, including Hubei (12.19%, aged 65–69) in 2012 [38] and Jilin (7.7%, aged 65–69) in 2014 [52]. Furthermore, previous study findings in other countries confirmed that an absence of adequate health information literacy is one of the most prevalent health issues for people of all ages, and older adults are considered as the largest group having limited health information literacy [14,15,16].

The present study findings indicate the role of the three socio-economic factors such as educational attainment, financial strain, and ethnicity in health information literacy, highlighting the effect of socio-economic inequality on health information literacy among older adults. As a result, the existence of a social gradient in health information literacy was confirmed in the current study, which extended the well-documented phenomenon of a social gradient in health domains [31,32,34,35,36] and enriched the applicable case of the theory of social determinants of health and social gradient in health [18,19,20,21,22,23,24,25,26].

Educational attainment was consistently related to health information literacy [13,51]; higher levels of education increased the likelihood of being health information literate among older adults. Educational attainment had a crucial effect on health information literacy, which may be because a knowledge gap results in an information gap [57] and a health information deficit. Growing evidence suggests that the public, especially patients, needs a higher education level and reading level as a means to support accessing and applying web-based health information in America [58]. Educational attainment contributed to knowledge and confidence in the ability to seek, understand, master, and use available health-related information among older adults in the US [59] and Finland [13].

Older adults from ethnic minorities were more likely to report lower health information literacy than Han Chinese. This may be because older adults of Han Chinese often have better supportive living, cultural, and language environments that contribute to more reading and better comprehension, while older adults from ethnic minorities have less opportunity to engage in supporting and enabling environments. This finding was similar to previous studies in the US [60,61], where the reasons for lower health information literacy lie in differences of culture and language. With the limitation of cultural background and a language barrier, older adults from ethnic minorities mainly depend on the oral tradition to obtain and disseminate information [62] and have less chance to attain and use healthcare-related resources.

Financial strain was an inhibitive predictor of health information literacy; an increase in financial strain resulted in a decline of health information literacy. Consistent with a previous study in Hubei province of Central China, it was demonstrated that a rise in individual income positively correlated with health information literacy [38]. With an increasing income and the basic physiological needs being met, older adults usually start to pursue developmental needs and are willing to pay more attention to healthcare and to take the initiative to access health information and healthcare services [63]. Therefore, older adults with better economic status tend to have more access, resources, and chances to obtain health information, which contributes to health information literacy.

In addition, these findings also highlighted the importance of other factors other than socio-economic status factors. Other factors including suffering from chronic diseases, seeking activity, reading magazines and books, and watching television were also significantly linked to health information literacy.

The experience of suffering from chronic diseases contributed to health information literacy. Consistent with previous studies, poor health was often treated as a reason for increased needs for health information and the seeking of it [43,44,45,46]; especially, having more chronic diseases increased the chances of seeking health-related information among older adults [47], which in turn played a positive role in health information literacy. It is well-known that those who suffer from chronic diseases are more concerned about negative outcomes of their diseases and thus are more likely to search for health information related to chronic diseases; this can keep patients informed about their chronic diseases—as the old Chinese saying goes: “prolonged illness makes the patient a good doctor” [64]. Conversely, some interesting findings are that those who have poor health seem to be more discouraged to seek and use information [13]; furthermore, patients with disease burdens often have a lower capacity to understand and use health information [65].

This study identified that those who were more active at seeking health information were more likely to be adequate in health information literacy. This finding is consistent with a previous study in Finland [13]. Being more active at information-seeking activity means that positive health-related motivations, beliefs, attitudes, and behaviors exist among older adults. Specifically, those who are more active at obtaining health information are also more confident in their ability to access, understand, evaluate, and apply health information. Hence, seeking activity can act as an enabler [66] to better health-related decision making.

Another finding of the current study was that magazines, books, and television as enabling factors enhanced health information literacy. Those who got health information from magazines, books, and television had more chances to improve health information literacy. This finding is similar to previous studies in the US, which revealed that books and magazines as a source of health information had an impact on health literacy [67], and television and magazines as a common delivery mechanism for health-related information can regularly provide more health promotion and disease prevention programs or articles for older adults [68]. Moreover, previous studies found that the Internet was independently associated with health information literacy [69,70]; however, the relationship between the Internet and health information literacy was not confirmed in the current study. Meanwhile, the impact of other health information sources including healthcare practitioners, family members, community workers, marketing staff, newspapers, and radio/broadcast on health information literacy was not confirmed in the current study. In China, magazines, books, and television are the commonly used sources of health information for older adults, and older adults adopt and use magazines, books, and television with fewer barriers, greater intentions, and higher frequencies. This may be because older adults are likely to extract more benefit from magazines, books, and television due to the better accessibility and availability.

## 6. Implications and Limitations

Due to the deficit and social gradient in health information literacy, this study implied that the improvement and reinforcement of health information literacy must be taken into account when developing public health strategies and actions to reduce health inequality among urban older adults in Western China. First, the current results suggest that health information literacy can be promoted by improving socio-economic status, such as educational attainment and economic conditions. Efforts must be made by providing lifelong education, health education, and financial support to strengthen an individual’s knowledge and capacity to make good health decisions. Second, national health planners and policymakers must redesign user-friendly and user-involving systems [71]. Health practitioners should encourage older adults to actively participate in information-seeking activity for health-related information. Furthermore, the accessibility and availability of health information sources such as magazines, books, and television should be improved to better guide, facilitate, and empower older adults in China.

The current study had several limitations. First, because of the limited human and financial resources, field testing for the health information literacy survey was limited to three provinces; the survey was conducted in only 3 of 12 western member areas in China, and the sample size was restricted to 812 respondents in total. Second, the cross-sectional design of this study prevents the causal associations between various predictors and health information literacy among older adults; future studies can employ a stronger research design, such as longitudinal studies, to establish a determination of cause and effect. Finally, occupation may be a very important socio-economic status indicator; however, the mechanism connecting occupation and health information literacy was not fully identified. Based on the standard occupational classification system, future studies can develop or introduce a new measurement of occupation to further examine the relationship between the two.

## 7. Conclusions

Consistent with previous studies, the current findings indicate the deficit and social gradient in health information literacy among urban older adults in Western China. This study demonstrates the crucial impact of socio-economic status on health information literacy. In addition, this study has theoretical implications that confirm and extend the well-documented phenomenon of social gradient in health information literacy in the Chinese context and that enrich the applicable case of the theory of social determinants of health and social gradient in health. Moreover, reducing the health information literacy deficit and social gradient in health information literacy must be considered as an important priority when developing public health and health education strategies, programs, and actions among urban older adults in Western China.

## Figures and Tables

**Table 1:** Characteristics of the sample by health information literacy level.4.1.

Characteristics	TOTAL	Health Information Literacy Level		χ2
		Inadequate	Adequate	
	*n* = 812	*n* = 676	*n* = 136	
	%	%	%	
Health information literacy level				
Inadequate	83.3	100.0	-	-
Adequate	16.7	-	100.0	
Gender				
Male	46.7	83.4	16.6	0.008
Female	53.3	83.1	16.9	
Age				
60–64	27.2	80.9	19.1	11.263 *
65–69	23.1	80.2	19.8	
70–74	23.0	83.9	16.1	
75–79	15.1	82.0	18.0	
80+	11.7	94.7	5.3	
Educational attainment				
Illiterate	20.3	98.2	1.8	78.669 ***
Primary school	30.4	91.1	8.9	
Junior high school	29.1	76.3	23.7	
Polytechnic school or senior	14.3	66.4	33.6	
College and above	5.9	66.7	33.3	
Ethnicity				
Han	84.2	81.6	18.4	8.703 **
Minority	15.8	92.2	7.8	
Occupation				
Ordinary staff	6.9	71.4	28.6	38.714 ***
Professional	19.0	62.5	37.5	
Manager	1.8	76.5	23.5	
Service industry employee	8.0	66.3	33.7	
Production staff	57.3	88.1	11.9	
Other	7.0	86.1	13.9	
Financial strain				
More than enough	5.9	70.8	29.2	44.477 ***
Good enough	35.3	73.9	26.1	
Approximately enough	41.1	87.7	12.3	
Somewhat difficult	12.9	95.2	4.8	
Very difficult	4.7	97.4	2.6	
Self-rated health				
Very bad	2.0	87.5	12.5	9.66 *
Bad	11.6	87.2	12.8	
Fair	34.5	86.8	13.2	
Good	40.4	78.4	21.6	
Very good	11.6	85.1	14.9	
Suffering from chronic diseases				
No	16.4	88.7	11.3	3.414
Yes	83.6	82.2	17.8	
Seeking activity				
Not at all	34.1	96.0	4.0	49.228 ***
Active	65.9	76.6	23.4	
Sources of health information				
Healthcare practitioners				
No	13.7	90.1	9.9	4.313 *
Yes	86.3	82.2	17.8	
Family members				
No	45.3	85.6	14.4	2.658
Yes	54.7	81.3	18.7	
Community workers				
No	82.5	84.5	15.5	4.133 *
Yes	17.5	77.5	22.5	
Marketing staff				
No	96.2	83.4	16.6	0.157
Yes	3.8	80.6	19.4	
Newspapers				
No	81.0	86.8	13.2	30.951 ***
Yes	19.0	68.2	31.8	
Magazines and books				
No	92.2	85.7	14.3	41.906 ***
Yes	7.8	54.0	46.0	
Radio/Broadcast				
No	94.1	83.5	16.5	0.61
Yes	5.9	79.2	20.8	
Television				
No	44.1	91.3	8.7	30.051 ***
Yes	55.9	76.9	23.1	
Internet				
No	90.3	85.7	14.3	31.751 ***
Yes	9.7	60.8	39.2	

Note: * *p* < 0.05, ** *p* < 0.01, *** *p* < 0.001.

**Table 2:** Binary logistic regression model of socio-economic status factors associated with health information literacy among urban older adults.

	Model I			Model II		
	B	Exp(B)	95% CI	B	Exp(B)	95% CI
Educational attainment	0.638 ***	1.893	1.537, 2.332	0.531 ***	1.700	1.332, 2.170
Ethnicity	−1.021 **	0.360	0.175, 0.742	−1.003 **	0.367	0.170, 0.790
Occupation	0.047	1.048	0.885, 1.241	0.023	1.024	0.853, 1.228
Financial strain	−0.454 ***	0.635	0.484, 0.833	−0.307 *	0.736	0.543, 0.997
Gender				0.113	1.120	0.728, 1.722
Age				−0.087	0.917	0.768, 1.095
Suffering from chronic diseases				1.082 **	2.951	1.466, 5.943
Self-rated health				0.012	1.012	0.777, 1.317
Seeking activity				1.025 **	2.787	1.392, 5.580
Sources of health information						
Healthcare practitioners				0.233	1.263	0.611, 2.612
Family members				0.073	1.075	0.696, 1.663
Community workers				0.502	1.651	0.980, 2.784
Marketing staff				−0.681	0.506	0.167, 1.530
Newspapers				0.352	1.422	0.878, 2.304
Magazines and books				0.949 **	2.582	1.345, 4.957
Radio/Broadcast				−0.744	0.475	0.200, 1.127
Television				0.529 *	1.698	1.033, 2.790
Internet				0.411	1.508	0.812, 2.801
Constant	−1.341	0.262		−4.986 ***	0.007	
−2LL	639.486			575.147		
Cox and Snell *R*^2^	0.11			0.177		

Note: * *p* < 0.05, ** *p* < 0.01, *** *p* < 0.001.

## Data Availability

The data presented in this study are available on request from the corresponding author. The data are not publicly available due to privacy restrictions.
